# TDO2‐augmented fibroblasts secrete EVs enriched in immunomodulatory Y‐derived small RNA

**DOI:** 10.1002/jex2.73

**Published:** 2023-02-02

**Authors:** Alessandra Ciullo, Kiel Peck, Xaviar Jones, Shukuro Yamaguchi, Ashley Anne Morris, Arati Naveen Kumar, Liang Li, Jamie Lee, Rodrigo Miguel dos Santos, Eugenio Cingolani, Ahmed Gamal Ibrahim

**Affiliations:** ^1^ Smidt Heart Institute Cedars‐Sinai Medical Center Los Angeles California USA

**Keywords:** extracellular vesicles, inflammation, macrophages, myocardial infarction, small RNA, TDO2, tryptophan 2,3‐dioxygenase, Y RNA

## Abstract

Mounting evidence implicates extracellular vesicles (EVs) factors as mediators of cell therapy. Cardiosphere‐derived cells are cardiac‐derived cells with tissue reparative capacity. Activation of a downstream target of wnt/β‐catenin signalling, tryptophan 2,3 dioxygenase (TDO2) renders therapeutically inert skin fibroblasts cardioprotective. Here, we investigate the mechanism by which concentrated conditioned media from TDO2‐augmented fibroblasts (TDO2‐CCM) exert cardioprotective effects. TDO2‐CCM is cardioprotective in a mouse model of MI compared to CCM from regular fibroblasts (HDF‐CCM). Transcriptomic analysis of cardiac tissue at 24 h demonstrates broad suppression of inflammatory and cell stress markers in animals given TDO2‐CCM compared to HDF‐CCM or vehicle. Sequencing analysis of TDO2‐EV RNA demonstrated abundance of a small Y‐derived small RNA dubbed ‘NT4’. Purification of TDO2‐EVs by size‐exclusion chromatography and RNAse protection assays demonstrated that NT4 is encapsulated inside EVs. Consistently with TDO2‐CCM, macrophages exposed to NT4 showed suppression of the inflammatory and cell stress mediators, particularly p21/cdkn1a. NT4‐depleted TDO2‐CCM resulted in diminished immunomodulatory capacity. Finally, administration of NT4 alone was cardioprotective in an acute model of myocardial infarction. Taken together, these findings elucidate the mechanism by which TDO2 augmentation mediates potency in secreted EVs through enrichment of NT4 which suppresses upstream cell stress mediators including p21/cdkn1a.

## Graphical abstract

Activation of tryptophan 2,3‐dioxygenase in a therapeutically inert cell type triggers secretion of cardioprotective EVs. These EVs are laden with a Y‐derived small RNA which mediates the therapeutic effect through suppression of p21‐p16 pro‐senescent signalling in macrophages to attenuate inflammatory activation.

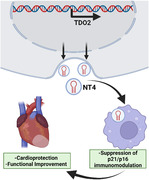

## 1 INTRODUCTION

Extracellular vesicles (EVs) are cell‐secreted lipid‐bilayer particles with an average diameter of 100–150 nm and contain a diversity of bioactive molecules, principally small non‐coding RNAs (Abramowicz & Story, 2020). Numerous lines of evidence demonstrate EVs are evolutionarily conserved modes of cellular communication and are critical in pathways of development and disease (Berumen Sánchez et al., 2021; Hur et al., 2021; Ibrahim & Marbán, 2016; Sahoo et al., 2021; Van Niel et al., 2022). EVs also mediate the therapeutic signalling of cell therapy (Cai et al., 2020; Ibrahim et al., 2014; Wiklander et al., 2019). EVs secreted from cardiosphere derived cells (CDC‐EVs) have demonstrated remarkable capacity to promote immunomodulation and tissue repair in several animal models of disease and injury (Ibrahim et al., 2014; Lin et al., 2021; Rogers et al., 2021). Systematic investigation has also identified monocyte‐derived macrophages as primary target cells of CDC‐EVs (De Couto et al., 2017, 2019; De Couto et al., 2015). The heterogenous composition of CDC‐EV molecular cargo contributes to their broad benefit but also makes mechanistic investigation challenging (Kalluri & Lebleu, 2020; Willis et al., 2017). Initial efforts in dissecting the mechanisms of therapeutic signalling implicated wnt/β‐catenin signalling as critical to the potency of CDCs and CDC‐EVs (Ibrahim et al., 2019).

Previous mechanistic investigations identified the metabolic enzyme tryptophan 2,3 Dioxygenase (TDO2) as a wnt/β‐catenin‐activated (Ibrahim et al., 2019). Augmenting skin fibroblasts (which have no therapeutic capacity) with TDO2 enhanced their therapeutic capacity (Ibrahim et al., 2019), suggesting an active role of TDO2 in wnt/β‐catenin therapeutic signalling in CDCs. Consistent with previous work, TDO2‐augmentation of fibroblasts endowed them and the EVs they secrete with potent immunomodulatory capacity of the macrophage response (Peck et al., 2021). Since cells exert their effect through the secretion of EVs, it became important to investigate the changes in EV cargo upon augmentation with TDO2. Here we dissect the payload of EVs from TDO2‐augmented fibroblasts (TDO2‐EVs) and the mechanism by which they signal to macrophages to attenuate inflammatory activation.

## 2 METHODS

### 2.1 Neonatal human dermal fibroblasts cells and reagents

Neonatal human dermal fibroblasts (HDF) were sourced from ATCC (PCS‐201‐010). Cells were cultured in IMDM (GIBCO), 10% FBS (Hyclone), 2 mM L‐glutamine (GIBCO) and gentamicin (GIBCO). Cells were maintained at 37°C 20% O_2_/5% CO_2_ in complete media with media exchanges every 3–4 days as needed. Cells were grown until near confluent and passaged using TrypLE (GIBCO).

### 2.2 Lentiviral transduction

HDFs were plated in T25 flasks and transduced with TDO2 (or empty vector control) activation lentiviral particles (MOI: 20, Santa Cruz Biotech) in complete media. Cells were transduced for 24 h, then media was replaced with fresh complete media for recovery, 24 h post‐recovery, selection was done using 5.0 μg/ml puromycin for 72 h. Cells were maintained thereafter in complete media.

### 2.3 Concentrated conditioned media preparation (CCM)

CCM were harvested from primary HDFs at passage 5–7, from normal and TDO2‐transduced cells using a 15‐day serum starvation method previously described (Walravens et al., 2021). Briefly, cells were grown to confluence in 20% O_2_ at 37°C. Cells were washed twice with warmed PBS to remove traces of complete media. Cells were conditioned in IMDM only for 15 days. Unless otherwise specified, CCM was prepared by 100 kDa ultrafiltration. Confirmatory studies demonstrating that NT4 is incapsulated in EVs were done using EVs isolated by size‐exclusion chromatography (both methods are described in greater detail below).

### 2.4 Conditioned media concentration by ultrafiltration and EV quantification

Following the conditioning period, media was harvested and cleared from dead cell debris through centrifugation at 3000 × *g* for 10 min followed by sterile filtration using a 0.45 μm filter. EVs were purified using centrifugal ultrafiltration with a 100 kDa molecular weight cutoff filter (Sigma–Millipore) followed by buffer exchange using phosphate buffered saline, then stored at −80°C. EV preparations were analysed by nanosight tracking analysis (NTA) using the Malvern Nanosight NS300 Instrument (Malvern Instruments) with the following acquisition parameters: camera levels of 15, detection level less than or equal to 5, number of videos taken = 5, and video length of 30 s.

### 2.5 EVs isolation using size‐exclusion chromatography (SEC)

EVs were collected and prepared as described above. After 100 kDa ultrafiltration EVs were further purified using size exclusion chromatography (SEC) columns (SmartSEC Single EV Isolation System; System Biosciences Inc; catalog# SSEC200A‐1) per technical instructions. Briefly, 1.0 ml of concentrated EVs were added to each chromatographic column and incubated at room temp with rotation for 30–35 min. EVs were eluted from the column by centrifugation at 500 × *g*. To isolate RNA from the EV‐depleted fraction, RNA was isolated directly from the column using miRNeasy (Qiagen). EVs size and concentration were analysed by NTA as described above. Protein content of EV preparations was quantified using a BCA assay (Pierce). Non‐EV fraction was also collected for NT4 qPCR.

### 2.6 Protein quantification in CCM and SEC‐isolated EVs

CCM and SEC‐isolated EVs were resuspended in PBS and protein concentration was measured using a DC Protein Assay kit (Bio‐Rad).

### 2.7 Bone marrow‐derived macrophage culture and LPS stimulation

Bone marrow‐derived progenitor cells were collected from 3‐month‐old female Wistar Kyoto rats and differentiated into bone marrow‐derived macrophages (BMDM) by culturing with 20 ng/ml recombinant M‐CSF (Life Technologies). Briefly, whole bone marrow cells were collected via aspiration with ice‐cold PBS. Cells were filtered using a 70 μm cell‐strainer and centrifuged at 400 × *g* for 10 min at 4°C to pellet. The cell pellet was resuspended in 10 ml ACK buffer (GIBCO) for 30 s. ACK was quenched with IMDM + 10% FBS and cells were centrifuged as described above. Cells were resuspended in complete media; IMDM +10% FBS + 20 ng/ml M‐CSF and counted. Cells were seeded into 6‐well plates at 8.0e10 (Hur et al., 2021) cells/well, or equivalent. Cells were incubated at 37°C with 20% O_2_ and 5% CO_2_. Fresh complete media was exchanged on day 3 and cells monitored for confluence. Test compounds were administered once BMDM cultures reached ∼75% confluence. Serum concentration was reduced to 1% during assays to facilitate EV uptake. To examine the immunomodulatory activity of test articles, macrophages were pretreated (1 h) with concentrated conditioned media, RNA (formulated in transfection reagent; Dharmafect [Horizon Discovery]) then exposed to lipopolysaccharide (LPS) (10 ng/ml, Sigma) or control (basal media). Eighteen hours post LPS exposure, RNA was isolated from cells for downstream analysis.

### 2.8 RNA isolation and qPCR

Total cell RNA was isolated using the RNeasy Plus Mini Kit (Qiagen) according to the manufacturer's protocol. RNA from concentrated conditioned media or SEC‐isolated fractions were isolated using the miRNeasy Advanced Serum Plasma Kit (Qiagen). Total cell RNA was quantified using nanodrop and diluted using dH_2_O. Total EV RNA was quantified by Qubit (Thermo Fisher Scientific). Cellular RNA Reverse Transcription was performed using the High‐Capacity RNA‐to‐cDNA kit (Life Technologies) with 1 μg RNA per reaction. PCR reactions were performed on the QuantStudio 7 Flex Real‐Time PCR System (Applied Biosystems) using TaqMan Fast Advanced Master Mix (Life Technologies, cat# 4444556) and TaqMan primers. Each reaction was performed in triplicate. The gene expression assays used for this study are summarised in Table 1.

**TABLE 1:** Gene expression assays

Assay names	Species	Assay ID
HPRT1	Mouse	Mm03024075_m1
IL6	Mouse	Mm00446190_m1
NLRP3	Mouse	Mm00840904_m1
IL‐1b	Mouse	Mm00434228_m1
IL‐23a	Mouse	Mm01160011_g1
Cdkn1a	Mouse	Mm04205640_g1
Cdkn1b	Mouse	Mm00438168_m1
Cdkn1c	Mouse	Mm00438170_m1
HPRT1	Rat and Mouse	Rn01527840_m1
IL‐1b	Rat	Rn00580432_m1
IL‐6	Rat	Rn01410330_m1
Cdkn1a	Rat	Rn00589996_m1

Cq values for genes were normalized using mouse and rat hypoxanthine phosphoribosyl transferase 1 (HPRT1).

### 2.9 NT4 quantification

To measure NT4 in concentrated conditioned media and EV samples, RNA was isolated miRNeasy RNA Isolation kit (Qiagen) miScript II RT Kit (Qiagen) was used for reverse transcription and qPCR was performed using QuantiTect SYBR Green (Qiagen) on a QuantStudio 12K Flex system (Applied Biosystems), with QuantiMir universal reverse primer, NT4 forward primer (5′‐GGTCCGATGGTAGTGGGTTATCAG‐3′) and U6 forward primer (5′‐TGGCCCCTGCGCAAGGATG‐3′) for the housekeeping gene.

### 2.10 NT4 copy number quantification

To assess the copy number of NT4 in EVs, we generated a copy number curve using known concentrations of NT4 oligo. The NT4 qPCR has an efficiency of 0.9923, the SYBR Green dissociation curve shows a distinct single peak at the melting temperature in relevant concentrations, indicating the specificity of the primers.

### 2.11 RNase and proteinase protection assay

For RNase and proteinase protection assay RNase A (Qiagen, 100 mg/ml stock solution) was used at a final concentration of 5 μg/ml, Proteinase K (Qiagen, 20 mg/ml stock solution) was used at a final concentration of 0.1 mg/ml and Triton X‐100 was used at 10% v/v. Briefly, SEC‐isolated EVs (SEC, SmartSEC Single EV Isolation System; System Biosciences) were treated with RNAse A for 20 min at 37°C. Samples were further treated with 0.1 mg/ml Proteinase K for 20 min at 37°C. Samples were pre‐treated with Triton 10% for 20 min at room temperature, followed by RNase A/Proteinase K incubation. RNA was isolated using the urine EV RNA isolation kit (Norgen Biotek, cat# 47200). qPCR was performed to assess NT4 expression levels.

### 2.12 Cytokine protein array

Cytokine content of bone marrow derived macrophage lysate from HDF‐EV or TDO2‐EV‐treated cells was analysed using the Rat Cytokine Array G2 (RayBiotech) using 300 μg of protein per sample and following the manufacturer's protocol. The RayBio® Analysis Tool software was used for analyses. The median values for the fluorescent intensities after local background subtractions are used. Then the positive control signal intensities are used to normalise the signals. To normalise signal intensity data, one sub‐array is defined as ‘reference’ to which the other arrays are normalised.

The normalised values are calculated as follows: X(Ny) = X(y) * P1/P(y)

where, P1= mean signal intensity of POS spots on reference array

P(y) = mean signal intensity of POS spots on Array ‘y’

X(y) = mean signal intensity for spot ‘X’ on Array ‘y’

X(Ny) = normalised signal intensity for spot ‘X’ on Array ‘y’

### 2.13 ELISA

Cardiac Troponin ELISA (R&D Systems, Quantikine ELISA) was performed according to the manufacturer's protocol. Samples concentration for testing was 1.2 mg/ml.

## 3 TRANSCRIPTOMIC ANALYSIS

### 3.1 Library preparation and sequencing

Total RNA samples were assessed for concentration using a Qubit fluorometer (ThermoFisher Scientific, Waltham, MA) and for quality using the 2100 Bioanalyzer (Agilent Technologies, Santa Clara, CA). Library construction was performed using the QIASeq Stranded RNA Library kit (Qiagen, Hilden, Germany) with QIAseq FastSelect ‐ rRNA HMR Kit (Qiagen) for ribosomal RNA depletion. Library concentration was measured with a Qubit fluorometer and library size on a Bioanalyzer. Libraries were multiplexed and sequenced on a NovaSeq 6000 (Illumina, San Diego, CA) using 75 bp single‐end sequencing. On average, approximately 50 million reads were generated from each sample.

### 3.2 Data analysis

Raw sequencing data was demultiplexed and converted to FASTQ format by using bcl2fastq v2.20 (Illumina, San Diego, CA). Then reads were aligned to the GRCm38 reference genome (http://www.gencodegenes.org) using STAR (version 2.6.1)4 with default parameters. Gene expression was quantified by RSEM (version 1.2.28)5 to generate a raw count expression matrix with gene identities as rows and samples as columns. DESeq2 (version 1.26.0)6 was used to normalise the raw count expression and correct the batch effect. Unsupervised principal component analysis (PCA) was performed by FactoMineR (version 2.3)7 in R (version 3.6.3) to check the replicates consistency. Then, each gene was fitted into a negative binomial generalised linear model, and the Wald test was applied to assess the differential expressions between two sample groups by DESeq2. Benjamini‐ Hochberg procedure was applied to adjust for multiple hypothesis testing, and differential expression gene candidates were selected with a false discovery rate less than 0.05.

### 3.3 Animal study

All animal studies were conducted under approved protocols from the Institutional Animal Care and Use Committee at Cedars‐Sinai Medical Center.

### 3.4 Mouse acute myocardial infarction model

Acute myocardial infarction was induced in three‐month‐old male C57BL/6J mice as described previously (Ibrahim et al., 2014). Within 10 min of left anterior descending artery ligation (LAD), a total of 2 × 10 (Lin et al., 2021) EVs (or vehicle) were administered via 3 × 8 μl injections intramyocardially.

### 3.5 Echocardiography

Echocardiography was performed in the mouse model of acute myocardial infarction at 1‐day (baseline) and 21‐days after surgery using Vevo 3100 Imaging System (Visual Sonics) as described (Ibrahim et al., 2019). The average of the left ventricular ejection fraction was analysed from multiple left ventricular end‐diastolic and left ventricular end‐systolic measurements.

### 3.6 Rat ischemic‐reperfusion model with retro‐orbital injection

To induce ischemia/reperfusion (I/R) injury, female Wistar Kyoto rats were provided general anaesthesia, and then a thoracotomy was performed at the 4th intercostal space to expose the heart and left anterior descending coronary artery. A 7–0 silk suture was then used to ligate the left anterior descending coronary artery, which was subsequently removed after 45 min to allow for reperfusion for 20 min. All RNA treatments are formulated in a small RNA transfection reagent (Horizon Discovery) Vehicle (PBS+ Dharmafect only), TDO2‐EVs (5 × 10^10^ particles in 250 μl PBS), NT4 (0.15 μg/g‐body weight, in 250 μl PBS+ Dharmafect, or NT4‐Scramble (0.15 μg/g‐body weight, in 250 μl PBS+ Dharmafect) were injected into test animals via retro‐orbital vein injection. After 48 h the animals were sacrificed. Blood was collected immediately prior to euthanasia for serum testing and the heart removed intact post‐mortem for TTC staining.

### 3.7 TTC staining

Two days following the I/R injury, animals were euthanised by cervical dislocation. Then, hearts were harvested, washed in PBS, and then cut into 1‐mm sections from apex to base, above the infarct zone. Sections were incubated with 1% solution 2,3,5‐triphenyl‐2H‐tetrazolium chloride (TTC) for 30 min at 37°C in the dark, washed with PBS, and then fixed overnight at 4°C in 4% paraformaldehyde. Then, sections were imaged and weighed. The infarcted zones (white) were delineated from viable tissue (red) and analysed (ImageJ software). Infarct mass was calculated in the tissue sections according to the following formula: (infarct area/tot area)/section weight (mg).

### 3.8 Statistics

GraphPad Prism 9.0 (GraphPad Software) was used to analyse the data. A comparison of three or more groups was performed using two‐way or one‐way ANOVA followed by Sidak's *post hoc* multiple comparison test for paired groups. Comparison between two‐groups was done using unpaired two‐tailed student's *t*‐test with a 95% confidence interval. RNA Sequencing data was analysed for differential expression, fold change and unsupervised PCA using DESeq2 (Anders & Huber, 2010; Love et al., 2014).

## 4 RESULTS

### 4.1 Concentrated conditioned media from TDO2‐augmented fibroblasts preserves cardiac function and attenuates cardiac tissue damage in a model of acute myocardial infarction

**FIGURE 1:**
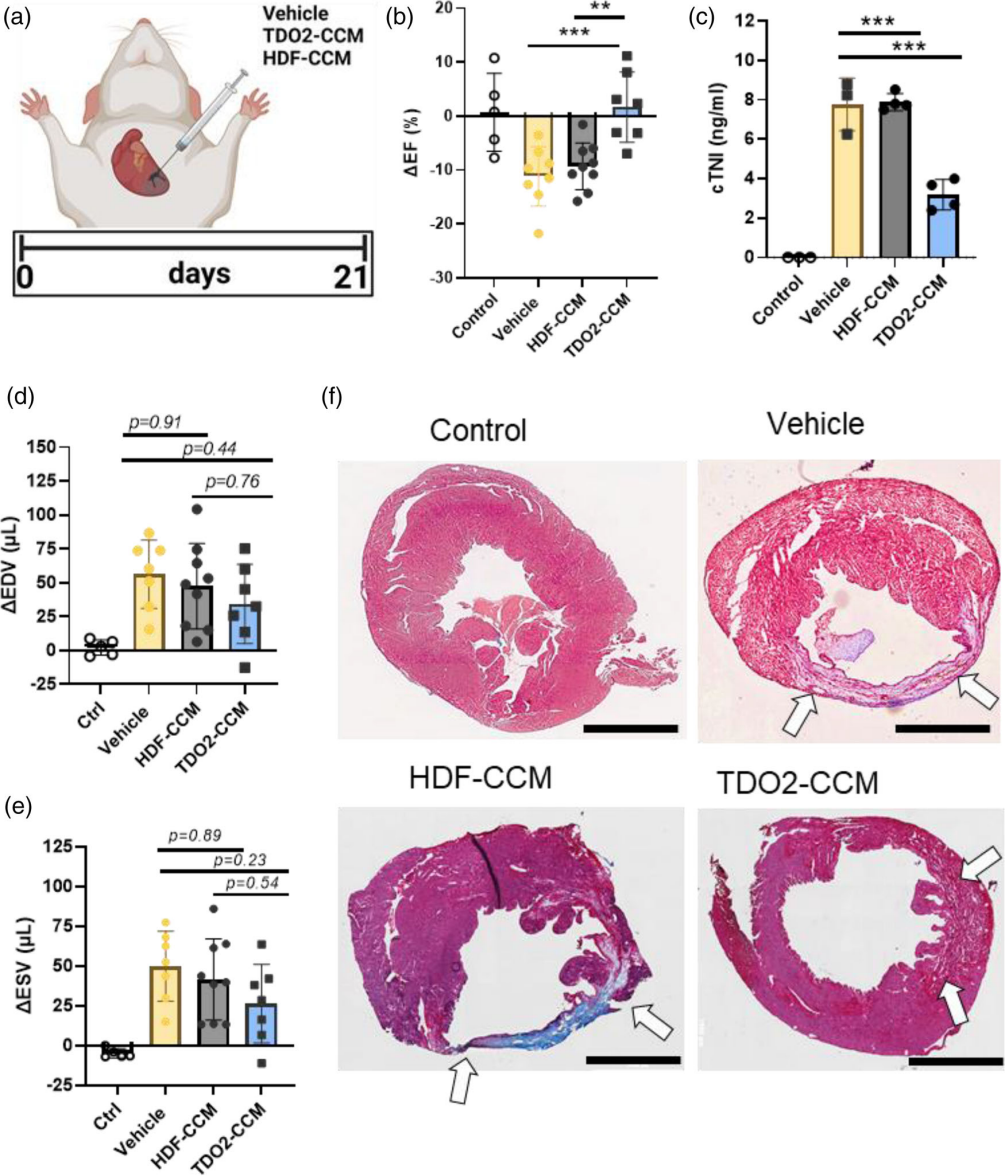
Concentrated conditioned media from TDO2‐transduced fibroblasts are therapeutic in a 3‐week mouse model of myocardial infarction. (a) Three‐week murine model of myocardial infarction. (b) Improved ejection fraction after 3 weeks when treated with TDO2‐CCM (*n* = 7–9 per group). (c) Cardiac Troponin I levels were significantly decreased at 24 h in animals treated with TDO2‐CCM, (*n* = 4 per group). (d,e) End diastolic and systolic volumes tended to be less in TDO2‐CCM treated hearts (*n* = 7–9 per group) though differences were not significant. (f) Masson's trichrome staining of left ventricles after 3 weeks (representative images; arrows point to scar tissue) indicates reduction in scar formation in TDO‐CCM treated hearts (scale bar: 2 mm). b–e were analyzed using one‐way ANOVA with Sidak's multiple comparison test. Error bars represent standard deviation,*p < 0.05, **p < 0.01, ***p < 0.001. biological replicates. (c–g) Analyzed using one‐way ANOVA with Sidak's multiple comparison test. Error bars represent standard deviation,*p < 0.05, **p < 0.01, ***p < 0.001

Having previously observed the potent immunomodulatory effects of TDO2‐augmented fibroblasts *in vivo* (Peck et al., 2021), and the immunomodulatory effects of TDO2‐EVs *in vitro* (Peck et al., 2021), we further examined whether concentrated conditioned media (from TDO2‐CCM) could recapitulate the disease‐modifying bioactivity of the engineered fibroblasts. TDO2‐CCM or CCM from and HDFs (HDF‐CCM) were prepared using ultrafiltration (100 kDa cutoff). The presence of EVs in these CCM samples was confirmed in previous work including the presence of conserved EV markers and absence of cellular contamination (Peck et al., 2021). We used a well‐established mouse model of acute myocardial infarction (AMI; Figure 1a). Animals with AMI given intramyocardial injections of TDO2‐CCM had improved cardiac function compared to those given HDF‐CCM (Figure 1b). Cardiac tissue damage, as measured by circulating cardiac troponin I levels (cTnI) were also significantly decreased in the TDO2‐CCM group versus either the HDF‐CCM or vehicle groups (Figure 1c). End diastolic and systolic volumes also tended to decrease in the TDO2‐CCM treated groups, though changes were not statistically significant (Figure 1d,e). Structurally, TDO2‐CCM‐treated hearts showed less remodelling compared to HDF‐CM‐treated hearts (Figure 1f). These results inspired further investigation of the mechanisms through which the TDO2‐fibroblast secretome was mediating these salutary effects.

### 4.2 TDO2‐CCM suppresses inflammatory and pro‐senescent markers 24 h post‐cardiac injury

**FIGURE 2:**
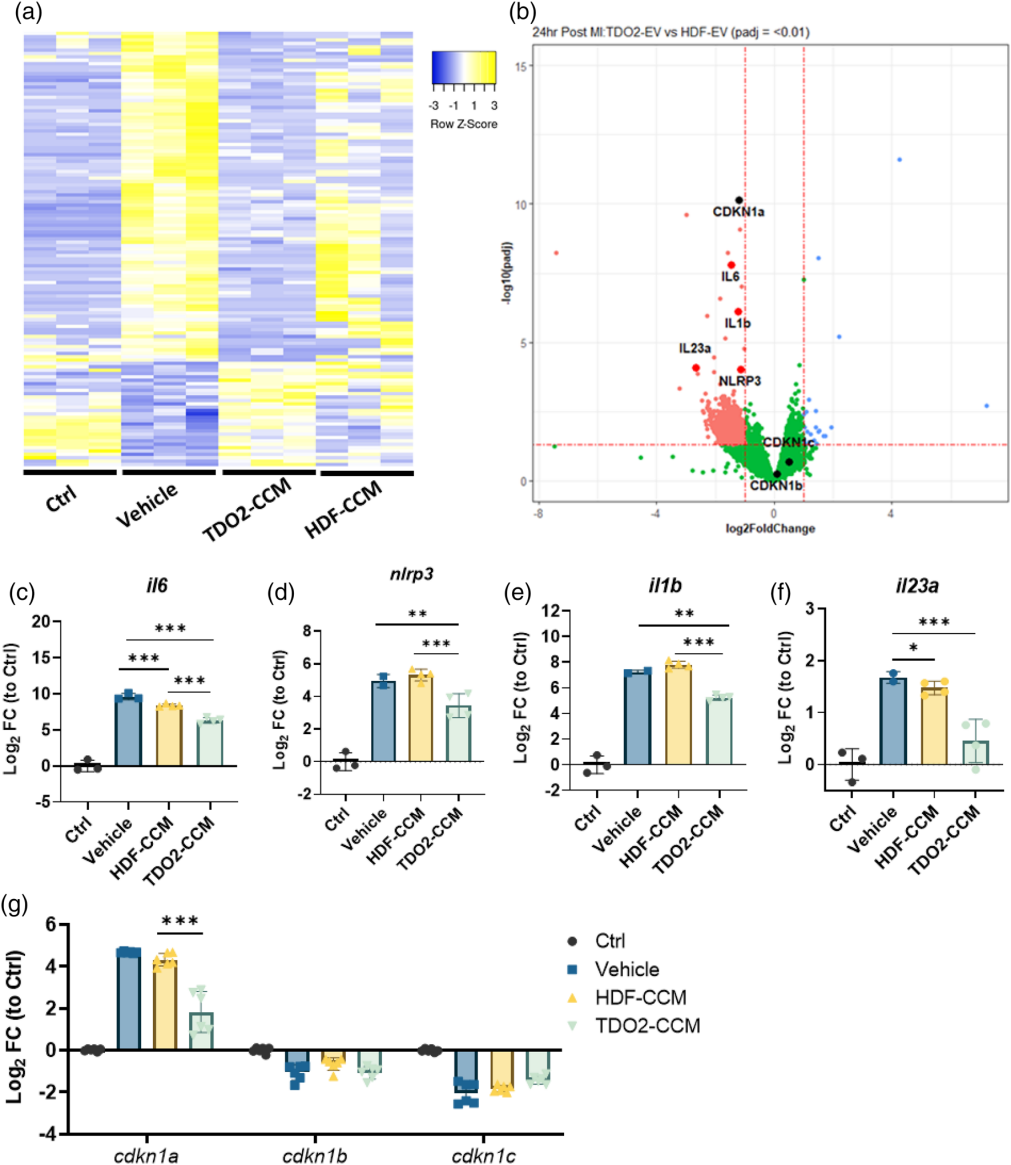
Sequencing of mouse cardiac tissues 24‐h post myocardial infarction and treatment reveals inflammatory regulation with TDO2‐EVs treatment. (a) Heat map of the top differentially expressed genes in left ventricle (partially including the infarcted region). (b) Volcano plot depicting the differential expression of genes when treated with TDO2‐CCM versus HDF‐CCM. Cyclin‐dependent kinase inhibitors 1a‐c and *il6*, *il1b*, *il23a* and *nlrp3* genes highlighted. (c–g) qPCR validation of differential expressed genes involved in the *cdkn1a*/*p21* cell senescence/inflammasome pathway, *n* = 3 biological replicates. (g) qPCR validation of *cdkn1a*, *cdkn1b* and *cdkn1c* expression in the infarcted region of the left ventricle 24‐h post‐MI, *n* = 3

In order to investigate cardioprotective effects of the TDO2‐HDF secretome, we isolated RNA from the infarct and border zone tissue from the groups described in Figure 1a, 24 h post‐injury. Transcriptomic analysis revealed broad restoration of gene expression (Figure 2a). In particular tissue injury markers including of inflammasome (*il1b* and *nlrp3*), and pro‐senescent (*cdkn1a*/*p21;* though other cyclin dependent kinase inhibitors including cdkn1b and cdkn1c remained unchanged) in infarcted hearts given TDO2‐CCM were suppressed compared to HDF‐CCM (Figure 2b). Suppression of these inflammatory mediators were further confirmed by qPCR including *il6*, *nlrp3*, *il1b* and *il23a* (Figure 2c–g). Down regulation of pro‐senescence signalling, suggests an immunomodulatory mechanism of action upstream of the previously hypothesised NF‐κβ (Peck et al., 2021).

### 4.3 Y‐derived small RNA, NT4, contained in TDO2‐EVs underlies the immunomodulatory properties of the TDO2‐augmented fibroblast secretome

**FIGURE 3:**
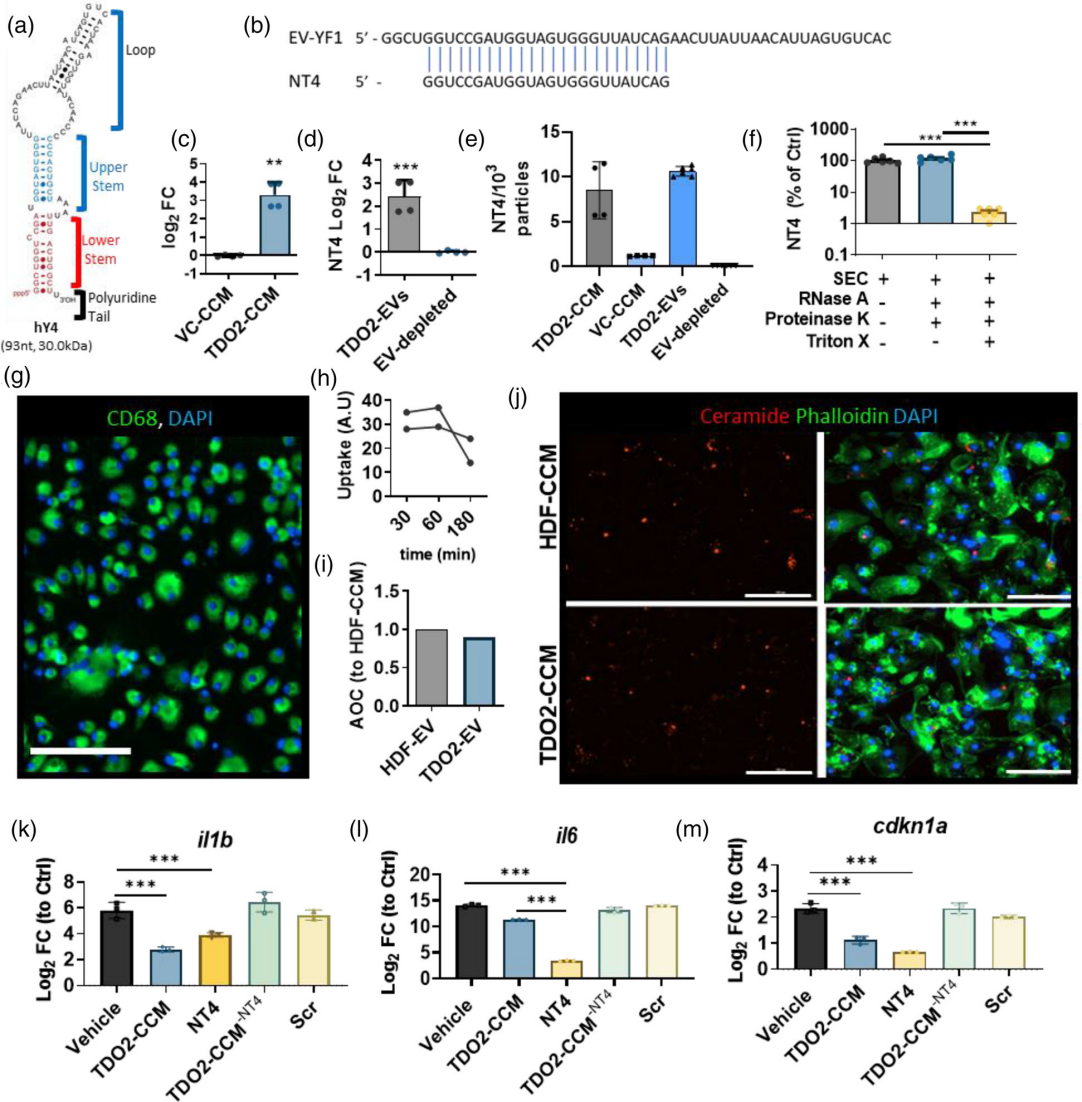
NT4 is a short non‐coding RNA fragment with immunomodulatory function derived from human Y RNA‐4 (hY4). (a) Schematic of the hY4 sequence and functional regions. (b) Sequence of NT4 and its position within the hY4 fragment EV‐YF1. (c) qPCR showing NT4 enrichment in CCM of fibroblasts transduced with TDO2‐activating lentivirus (TDO2‐CCM) compared to those from fibroblasts transduced with a lentivirus containing a control vector (VC‐CCM). (d) QPCR of NT4 from SEC‐isolated EVs compared to the EV‐depleted fraction. (e) Absolute quantification (NT4 copies/10^3^ particles) of TDO2‐CCM, VC‐CCM, SEC‐isolated TDO2‐EVs and the EV‐depleted fraction. (f) RNAse protection assay demonstrating NT4 is encapsulated inside TDO2‐EVs. (g) Broad expression of the monocyte marker, CD68, in bone marrow‐derived macrophages. (h) Equivalent uptake of TDO2‐ and HDF‐EVs (EV ceramide dye; red, phalloidin: green and nuclei: blue) by bone marrow derived macrophages at 30, 60 and 180 min post exposure as measured by internalisation of red signals inside macrophages and, (i) area under the curve measurement of cumulative signal at all three time points. (j) Representative image of TDO2‐ and HDF‐EVs (EV ceramide dye; red, phalloidin: green and nuclei: blue) by bone marrow derived macrophages at 1‐h post‐exposure. (k–m) TDO2‐EV and NT4 pre‐treatment lowered expression of inflammatory genes *il1b* and *il6*, as well as pro‐senescence *p21*/*cdkn1a*. Suppression of NT4 in TDO2‐CCM abrogated the suppressive effect of TDO2‐CCM on *il1b*, *il6* and *p21/cdkn1a* in LPS‐stimulated macrophages. Scale bars: 100 μm. Statistical analysis between two groups was done using Student's *T* test and comparisons between three or more groups was done using one‐way ANOVA with Sidak's multiple comparison test. Error bars represent standard deviation,*p < 0.05, **p < 0.01, ***p < 0.001

Previous work by our group implicated canonical Wnt signalling as a driver of cell and EV therapeutic potency. In particular, therapeutic EVs were enriched in fragments originating from the loop domain of the YRNA‐4 gene (Figure 3a). Previously described fragments include a therapeutically bioactive 56 nt fragment (dubbed EV‐YF1 (Cambier et al., 2017; Huang et al., 2021)) and a shorter variant (dubbed NT4 (Ciullo et al., 2022); Figure 3b). As TDO2 is a primary activation target of canonical Wnt signalling, we hypothesised that TDO2‐fibroblasts secreted EVs enriched in Y‐derived small RNA including NT4. In previous work we sequenced the RNA payload of TDO2‐EVs to broadly characterise the RNA classes present in TDO2‐EVs (Peck et al., 2021). Using CDC‐EVs as a positive control, we further observed that CDC‐EVs were more abundant in NT4 compared to TDO2‐EVs (Figure S2A). This be rationalised by the fact that CDCs which have the activation of more downstream genes of canonical Wnt signalling than TDO2 only to have higher levels of NT4. To investigate whether NT4 was EV‐derived, we used SEC. SEC‐isolated EVs had markedly lower protein content compared to CCM equivalents (Figure S2B,C) with no changes in particle numbers between the two preparations (Figure S2D). QPCR quantification of SEC‐isolated EVs demonstrates that NT4 is enriched in TDO2‐EVs compared to EVs from fibroblasts transduced with a lentivirus vector control (VC‐EVs; Figure 3c). Analysis of NT4 abundance in CCM and EVs isolated by size‐exclusion chromatography indicated that NT4 was enriched in the EV fraction and deficient in the EV‐depleted fraction (Figure 3d). Absolute quantification of NT4 copies using a copy number curve (Figure S2B) demonstrated that TDO2‐CCM contained nearly ten‐fold more copies of NT4 per EV particle compared to VC‐CCM (Figure 3e). Furthermore, NT4 is preferentially packaged in EVs as this abundance was conserved in the SEC‐isolated EVs while the EV‐depleted fraction was deficient in NT4 by comparison (Figure 3e). To further demonstrate that NT4 was encapsulated within the EVs, we performed an RNAse protection assay to demonstrate that NT4 was protected from RNAse activity unless the integrity of the EV membrane was compromised (Figure 3g). To investigate mechanism of NT4 signalling in target tissue we decided to focus on macrophages, which have been shown to be critical in cell, EV and small RNA therapy. To confirm that the BMDM we use are monocyte‐derived macrophages (BMDMs), we demonstrated that these cells broadly express CD68, a marker of the monocyte lineage (Figure 3h). Finally, though TDO2‐EVs are enriched in NT4, variable uptake efficiency may undermine this enrichment. To rule out the possibility, we stained CCM preparations with bodipy, (fluorescent ceramide stain) a commonly used EV dye. Analysis of EV‐uptake at 30 min, 1 h, and 3 h demonstrated comparable uptake (Figure 3i). Cumulative EV uptake as measured by the area under the curve further demonstrated comparable uptake of TDO2‐ and HDF‐EVs by macrophages (Figure 3j) and representative images (Figure 3k). Furthermore, NT4 suppressed pro‐inflammatory activation in LPS‐stimulated macrophages including *il1b* and *il6* and *cdkn1a/p21* (Figure 3k–m). These salutary effects were abrogated when TDO2‐CCM was depleted of NT4 by admixing TDO2 CCM with 80 nM of anti‐sense NT4 oligonucleotide (in transfection reagent; TDO2‐CCM^−NT4^) indicating that TDO2‐EV activity is mediated significantly by NT4 signalling (Figure 3l–n).

### 4.4 NT4 exerts broad anti‐inflammatory effects in LPS‐stimulated macrophages

**FIGURE 4:**
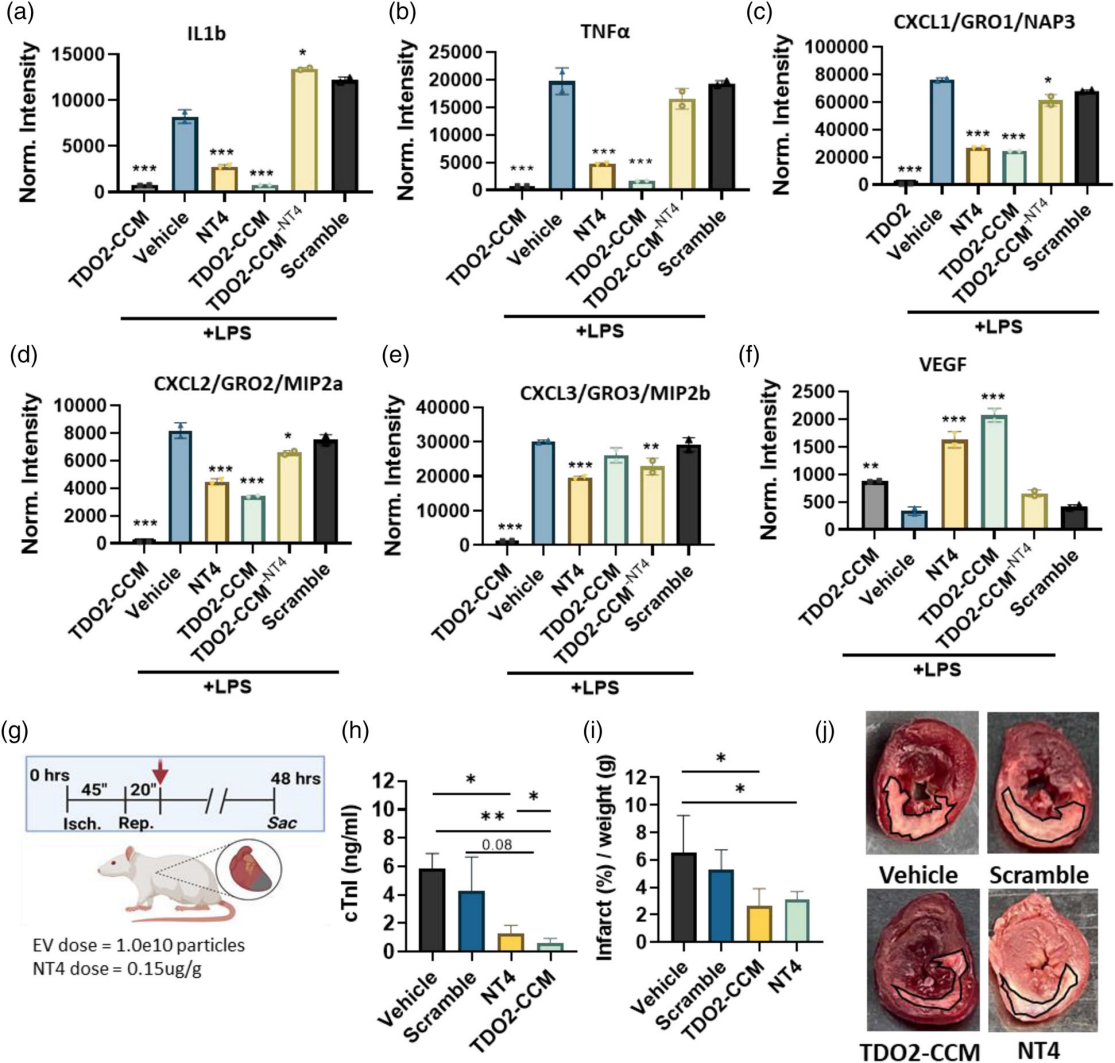
Inflammatory activation and recruitment in bone marrow derived macrophages is regulated by pre‐treatment with TDO2‐EV or NT4. (a–d) Protein array demonstrating that pre‐treatment of LPS‐activated macrophages with TDO2‐CM or NT4 resulted in decreased levels of the inflammatory mediators IL‐1b (a), TNFα (b) and the chemokines CXCL1 (c), CXCL2 (d) and CXCL3 (e). (f) The pro‐angiogenic mediator VEGF was also significantly upregulated in macrophages pre‐treated with TDO2‐EV or NT4. Depletion of NT4 in TDO2‐CCM diminished or entirely abrogated those effects (a–f; all statistical analyses are done in comparison to the vehicle group). (g) Schematic of the rat model of ischemic cardiac injury and reperfusion. (h) Serum levels of Cardiac Troponin I were significantly decreased in NT4 and TDO2‐EV treated animals compared to those receiving no treatment, *n* = 4. (i) Quantification of the percentage of infarct normalised to section weight, *n* = 5 animals/group. (j) Representative images of TTC staining (infarct region outlined in black). The white‐stained tissue is indicative of infarcted (dead and dying) tissue

To further investigate the immunomodulatory function of NT4, bone marrow derived macrophages were transfected with NT4, an NT4 scramble control, or TDO2‐CCM with an anti‐sense to NT4 prior to LPS exposure. Analysis of cell lysate using a cytokine array (Figure S1A–D) showed trend toward suppression of *il6* (Figure S1B) in NT4 and TDO2‐CCM compared to other groups. Furthermore, both NT4 and TDO2‐CCM significantly reduced the inflammatory cytokines *il1b* and *TNFα* (Figure 4a,b), and the monocyte recruitment chemokines CXCL1, 2 and 3 (Figure 4c–e, respectively). Finally, both NT4 and TDO2‐CCM increased the expression of the pro‐angiogenic mediator, vascular endothelial growth factor (VEGF; Figure 4f) compared to other groups. Macrophages exposed to TDO2‐CCM^−NT4^ did not exhibit the immunoregulatory or pro‐angiogenic phenotype observed with either NT4 of TDO2‐CCM (Figure 4a–f). Interestingly, some differences were observed between TDO2‐CCM and NT4. For instance, TDO2‐CCM and TDO2‐CCM‐NT4 groups had diminished levels of the anti‐inflammatory cytokine *il10* compared to vehicle while NT4 had slightly higher levels (though this trend was not statistically significant; (Figure S1C). Furthermore, TDO2‐CCM alone suppressed pro‐inflammatory *il1a* levels compared to other groups (Figure S1D). This indicates that other components of TDO2‐CCM or, more specifically EVs, mediate effects beyond NT4 signalling.

### 4.5 NT4 is cardioprotective in a 48‐h rat model of ischemia reperfusion injury

Given that NT4 pre‐treatment leads to immunoregulation, we sought a short‐term model cardioprotection model. Therefore, we investigated the therapeutic bioactivity of NT4 in a rat ischemia reperfusion injury model (Figure 4g). Female Wistar Kyoto rats were given a myocardial infarct through temporary ligation of the left atrial descending coronary artery. Following reperfusion animals were given vehicle, NT4‐scramble, TDO2‐EVs or NT4 intravenously (retroorbital delivery). Results of circulating cTnI levels 48 h post reperfusion indicated attenuation of myocardial damage in NT4 and TDO2‐EV treated animals compared to other groups (Figure 4h). This observation was further reinforced using TTC staining showing reduced infarct sizes (white area of the tissue) from NT4 or TDO2‐CM‐treated groups were less than that from the vehicle group (Figure 4i,j).

## 5 DISCUSSION

Dissecting the various pathways implicated in the mechanisms of action of cell therapy (and their effector EVs) is a fundamental pillar of translation in regenerative medicine. Previous work has shown that CDCs are a therapeutic cell type with the capacity to repair cardiac and other tissue after injury (Marbán, 2018). Among the pathways that drive their therapeutic efficacy is the canonical wnt/β‐catenin signalling (Ibrahim et al., 2019). CDCs, like other cell therapies impart their therapeutic function through the secretion of EVs (Barile et al., 2014; Hirai et al., 2020; Ibrahim et al., 2014; Peck et al., 2021). EVs work by delivering bioactive molecules like small non‐coding RNAs with salutary and pleotropic effects. These include gene‐regulation in injured tissue, notably through modulation of the macrophage response (De Couto et al., 2017, 2019; Peck et al., 2021). The work detailed here represents a continuation of this mechanistic elucidation. Building from previous findings that TDO2 is a major upregulated target of wnt/β‐catenin, we demonstrate that augmenting TDO2 expression in dermal fibroblasts switches their phenotype from therapeutically inert to potently immunomodulatory (Peck et al., 2021). In an *in vivo* model of acute myocardial infarction, we confirmed that the secretome of TDO2‐augmented EVs exerted therapeutic effects compared to the secretome of normal fibroblasts. Early timepoint sequencing of TDO2‐CCM‐treated injured hearts reveal potent immunomodulatory activity as demonstrated by down‐regulation of inflammatory genes. Furthermore, we found a suppression of the pro‐senescent gene *cdkn1a /p21* which provides evidence in support a more upstream level of inflammatory regulation. To elucidate the effects of TDO2 augmentation on secreted EV contents, we mined EV cargo for therapeutically relevant constituents. Among the most notable differences was NT4. NT4 subsequently demonstrated immunomodulatory capabilities similar to those seen in the TDO2‐EVs including down‐regulation of inflammatory genes *il1b*, *nlrp3* and *il6*, as well as suppression of the pro‐apoptotic *p16*/*p21* senescent genes. Further interrogation of NT4‐treated macrophages demonstrated a broad down regulation of inflammatory markers at the protein level as well. Analysis of cell lysates from NT4 treated macrophages show suppression of inflammatory initiator *il1b* and *TNFα*, as well as down‐regulation of *il6* and the *cxcl1*‐*3* cytokines. *Cxcl1* and *cxcl2* are both members of the chemokine family and are responsible for the recruitment of neutrophils and circulating macrophages to the site of injury (De Filippo et al., 2013). *Cxcl3* along with its receptor *cxcr2* is mainly responsible for monocyte migration and adhesion signals and have been reported to be overexpressed in malignant cancer types (Sun et al., 2022). Consistent with gene expression changes, proteomic analysis of NT4‐treated macrophages revealed suppression of pro‐inflammatory cytokines and chemokines including *il1b*, TNFα and *cxcl1‐3* (respectively) and increased pro‐angiogenic cytokines including VEGF. *In vivo*, NT4 administration led to a decrease in serum levels of cTnI and a reduction in infarct size in a model of ischemia/reperfusion injury. These two readouts indicate cardioprotection, likely caused by attenuation of pro‐inflammatory and pro‐senescence pathways. Taken together these data strongly indicate that the mechanism of action for therapeutically competent TDO2‐EVs is due to expression of the pleotropic YsRNA NT4.

### 5.1 Study limitations

Despite the findings presented here, many questions remain unanswered and the focus of ongoing work. For instance what is the mechanistic link between β‐catenin (and TDO2) activation and the transcription of YsRNAs like NT4? How does NT4 regulate the gene targets described here? What is/are the putative binding partner(s) of NT4? It is important to note that YRNA4 is not conserved in mice and therefore, its ability to exert transcriptome regulating functions is an area of active investigation by our group. One possible explanation is that the genes that NT4 affects retain sufficient homology with the human transcriptome that retains the signalling capability of this small RNA in the murine genome. Furthermore, given that NT4 acts upon tumour suppressor pathways, what is the safety profile of repeated administration in an immune compromised model (i.e., tumorigenicity)? What is the biodistribution profile of IV‐administered NT4? How long does its bioactivity last? Given the inherent differences in the delivery efficiency and profile of synthetic NT4 encapsulated in a transfection reagent and NT4 encapsulated in EVs, a direct comparison of the therapeutic capacity of the two treatments is difficult. However, the diminished immunomodulatory activity of NT4‐depleted TDO2 EVs suggests that NT4 plays an active role in TDO2‐EV signaling. Future studies will also examine the sufficiency and necessity of macrophage signalling in NT4 bioactivity *in vivo*. The translational implications of a small RNA having pleotropic effects against inflammatory, and senescence are not small. Such a therapeutic could be administered both in an acute interventionist fashion as well as alongside premeditated procedures such as cardiac artery stenting and bypass.

## 6 CONCLUSIONS

We found that TDO2 activation in HDF modulates the secretion of cardioprotective EVs, enriched in a Y‐derived small RNA, NT4, which exerts its immunomodulatory effects, at least in part, through suppression of the p21‐p16 pro‐senescence pathway.

## AUTHOR CONTRIBUTIONS

Alessandra Ciullo: Data curation; Formal analysis; Project administration; Writing – review & editing. Kiel Peck: Data curation; Formal analysis; Investigation; Methodology; Project administration; Writing – original draft; Writing – review & editing. Xaviar Jones: Investigation; Methodology. Shukuro Yamaguchi: Investigation. Ashley Anne Morris: Investigation. Arati Naveen Kumar: Investigation; Methodology. Liang Li: Methodology; Project administration. Rodrigo Miguel dos Santos: Investigation; Methodology. Jamie Lee: Investigation; Methodology. Eugenio Cingolani: Conceptualization. Ahmed Gamal Ibrahim: Conceptualization.

## Supporting information

Supporting Information
