# Supplementary material for: TDO2‐augmented fibroblasts secrete EVs enriched in immunomodulatory Y‐derived small RNA
Source: J Extracell Biol. 2023 Feb 2;2(2):e73. doi: 10.1002/jex2.73 (PMC11080885; doi:10.1002/jex2.73)
Supplement: Supplementary file 1 — Supporting Information [file JEX2-2-e73-s001.docx]

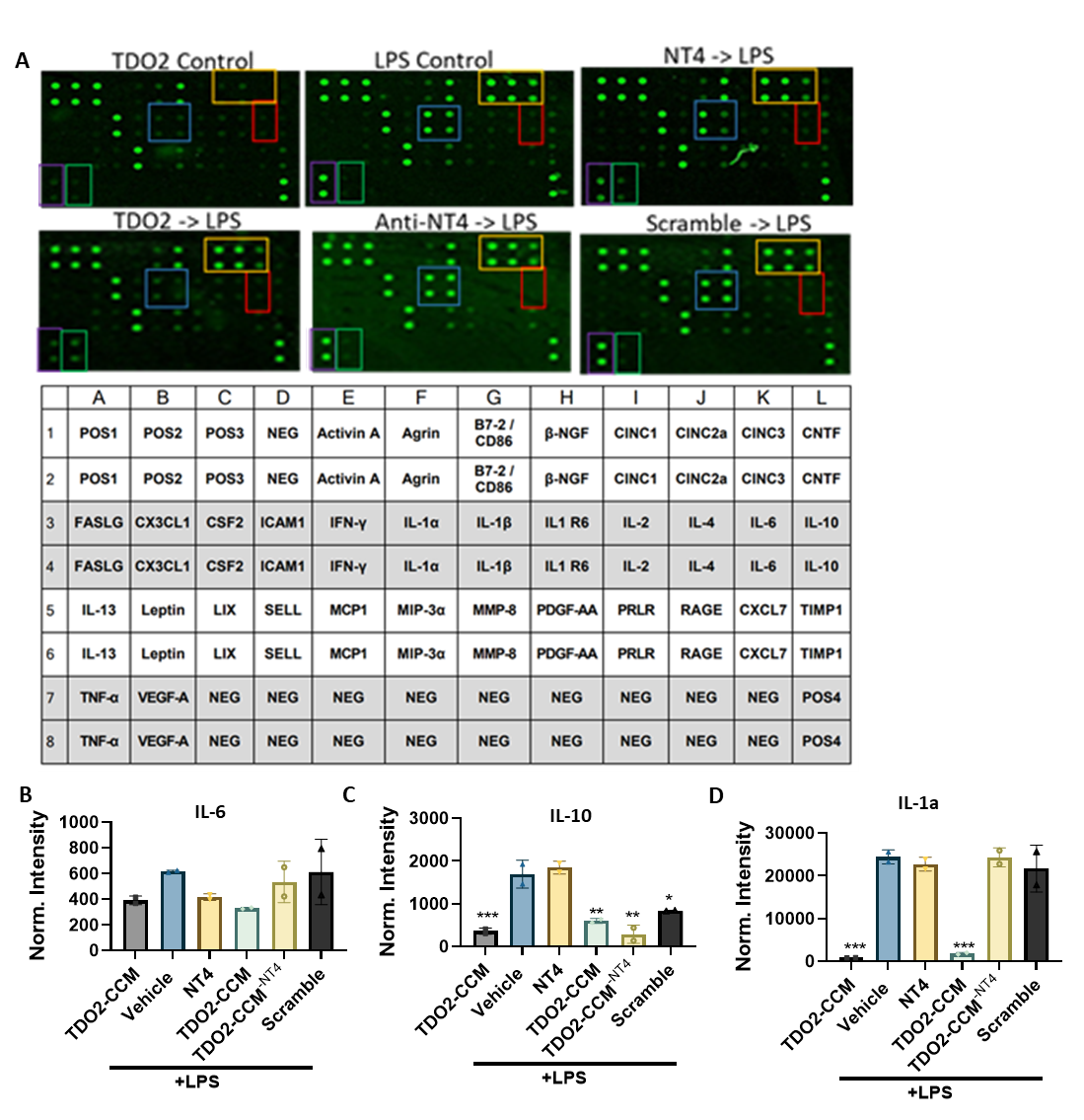
Supplementary Data

**Supplementary Figure 1. A.** Rat Cytokine array snapshot with table of samples. Scanned results for rat cytokine array. Graphed areas highlighted. Yellow = CXCL1, CXL2, and CXCL3, Red = IL-6, Blue = IL-1a and IL-1b. Purple = TNFa, Green= VEGF-A **C** Array map for RayBiotech Rat Cytokine Array G2. CINC1 = CXCL1 (human GRO-α homolog), CINC2a = CXCL3 (human GRO-γ homolog), CINC3 = CXCL2 (human GRO-β homolog), FASLG = Fas Ligand, CX3CL1 = Fractalkine, CSF2 = GM-CSF, LIX = CXCL5, SELL = LSelectin, PRLR = Prolactin Receptor, RAGE = Receptor for Advanced Glycosylation Endproducts, CXCL7 = Thymus Chemokine 1. VEGF-A detects VEGF(165 aa) and VEGF(121 aa). (**B-D**) Protein array of LPS-activated macrophages pre-treated with preparations of TDO2-CCM, NT4, or scramble. Data analyzed using One-Way ANOVA (all groups compared to vehicle) with Sidak’s post test for multiple comparisons. All error bars represent standard deviations; *p<0.05, **p<0.01, ***p<0.001.


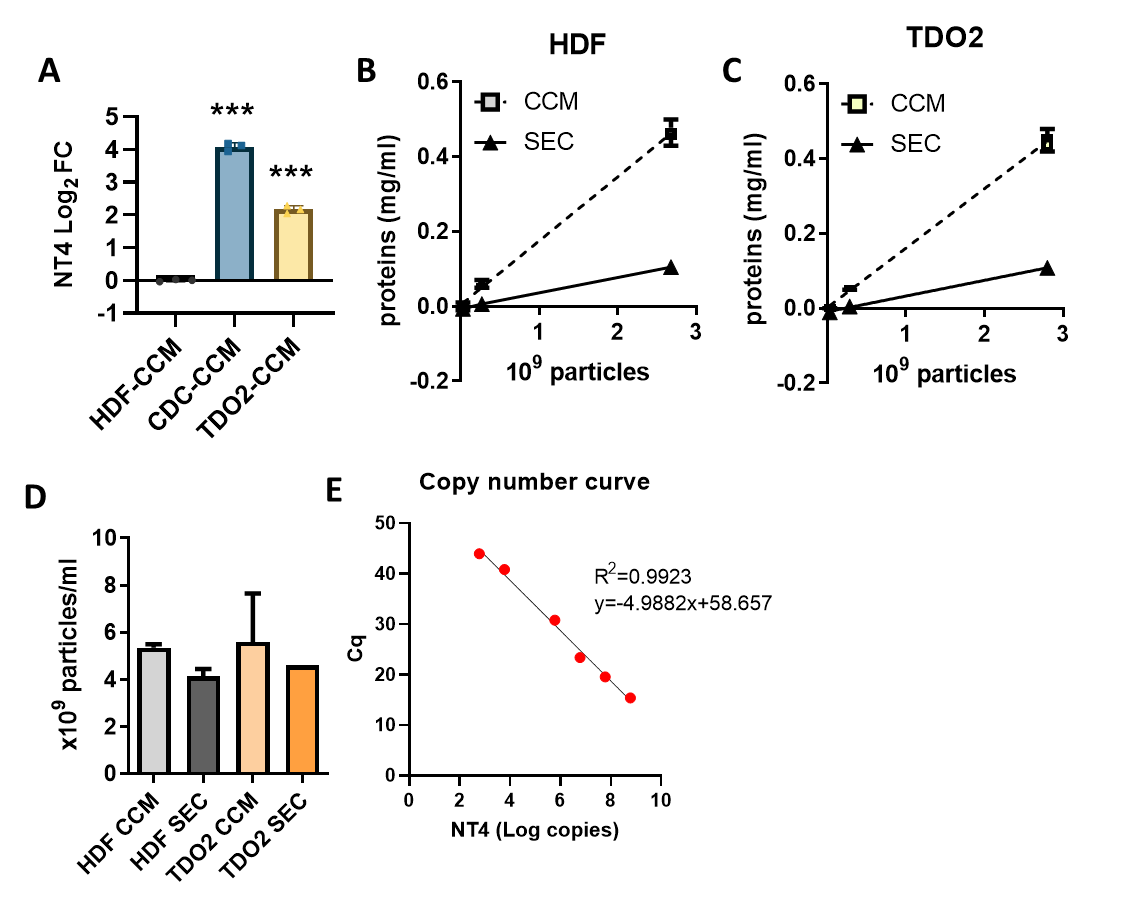
**Supplemental Figure 2. A.** QPCR of NT4 expression in the CCM of HDF, CDC, or TDO2-HDFs. **B, C.** Protein content in particles from concentrated conditioned media and size-exclusion chromatography-isolated EVs (at three different EV dilutions). **D.** Particle concentration from concentrated conditioned media and size-exclusion chromatography preparations. **E.** Copy number curve of NT4 copy numbers and corresponding Cq values by qPCR. Bar graphs and error bars represent mean and standard deviation respectively. Data was analyzed using One-Way ANOVA (all groups compared to vehicle) with Sidak’s post test for multiple comparisons. All error bars represent standard deviations; *p<0.05, **p<0.01, ***p<0.001.
